# Advanced Surface Ablation in a Patient With Suspect Topography: A Case Report

**DOI:** 10.7759/cureus.60277

**Published:** 2024-05-14

**Authors:** Rodrigo Vilares Morgado, Jaime Guedes, Ana Margarida Ferreira, Marcella Q Salomão, Fernando Faria-Correia, Amândio Rocha Sousa, Renato Ambrósio

**Affiliations:** 1 Department of Ophthalmology, Centro Hospitalar Universitário de São João, Porto, PRT; 2 Department of Glaucoma, Wills Eye Hospital, Philadelphia, USA; 3 Department of Ophthalmology, Rio de Janeiro Corneal Tomography and Biomechanics Study Group, Rio de Janeiro, BRA; 4 Department of Ophthalmology, Hospital de Braga, Braga, PRT; 5 Department of Ophthalmology, Federal University of the State of Rio de Janeiro, Rio de Janeiro, BRA

**Keywords:** photorefractive keratectomy, surface ablation, keratoconus suspect, keratoconus, corneal ectasia

## Abstract

The purpose of this clinical report is to describe a 10-year clinical outcome of advanced surface ablation with photorefractive keratectomy (PRK) in a patient who had been previously incorrectly diagnosed with keratoconus (KC). Corneal ectasia is a rare but extremely relevant complication of laser vision correction, and KC represents a major contraindication for these procedures. Nonetheless, some surface ablation procedures, such as PRK, might be a valid option for particular patients with atypical corneal topography or subclinical or mild forms of KC. Patient education and complete preoperative refractive multimodal imaging are essential for a more conscious therapeutic decision, minimizing iatrogenic ectasia, as well as decreasing the number of patients who are incorrectly denied refractive surgery, as was the patient presented in this study.

## Introduction

Keratoconus (KC) is the most frequent type of corneal ectasia. It is bilateral, though frequently asymmetric, and usually presents in the second to third decade of life. This disease is characterized by corneal stromal thinning, accompanied by an apical protrusion (ectasia), with consequent increased corneal curvature, irregular astigmatism, increased higher-order aberrations (HOA), and mild to significant visual impairment [1,2]. It has a reported prevalence of about 1/2000 in the general population, depending on the population described, but in patients aged 10-40 years, its prevalence can be as high as 1/375, and more recent studies suggest a prevalence that can be as high as 4.79% in general pediatric patients [3-5]. The prevalence may vary depending on the diagnostic criteria used and the ethnicity of the study population. For many patients with KC, an acceptable improvement in visual acuity can be achieved using refractive correction with spectacles or contact lenses (CL). However, in some cases, the quality of vision might still be reduced due to corneal optical aberrations due to irregular astigmatism associated with the disease [6,7].

Keratorefractive surgery, particularly laser in situ keratomileusis (LASIK), has traditionally been contraindicated in these patients due to the risk of iatrogenic postoperative progression of the disease [6,7]. The creation of a lamellar flap weakens the corneal biomechanical properties, which, in cases of KC, is already susceptible and can lead to a higher risk of ectasia progression [8]. A fundamental concern when performing corneal laser vision correction (LVC) in eyes with atypical corneal topography is the topographic progression of the ectatic disease that may occur following the procedure, compromising uncorrected distance visual acuity (UDVA) and corrected distance visual acuity (CDVA), rendering the treatment not only ineffective but also deleterious to the patient and their vision-related quality of life (VRQOL) [9]. Risk factors for the development of post-LVC ectasia or increased KC progression include the following: (1) young age; (2) personal or family history of KC; (3) high myopia; (4) abnormal preoperative topography/tomography; (5) low residual stromal bed (RSB) thickness (<300 µm); (6) excessive stromal ablation (>100 µm); (7) low preoperative central corneal thickness (CCT < 500 µm); (8) high percentage of tissue altered (PTA > 0.40); and (9) deep primary keratotomy resulting in a thick corneal flap [9-11]. However, multiple long-term studies report the safety of surface ablation procedures in milder forms of KC (which include subclinical and forme fruste KC) [7,12-14]. Recent therapeutic options with photorefractive keratectomy (PRK) in cases of mild forms of KC use topography-guided ablation profiles, which aim to improve the regularity of the corneal surface and thus enhance the quality of the patient’s vision [13]. Although iatrogenic cases of corneal ectasia have been previously described after PRK in KC suspects [15], several other studies have reported positive and encouraging results [14,16].

This case report aims to describe the 10-year clinical outcome of a patient who was initially misdiagnosed with KC and denied the possibility of undergoing corneal LVC, who eventually was re-evaluated with multimodal corneal and anterior segment imaging and underwent advanced surface ablation in both eyes (OU). The patient’s corneal topographic, tomographic, and biomechanical properties have been stable for 10 years. The patient in this case report signed an informed consent form to authorize its publication.

## Case presentation

In 2013, a 35-year-old male patient presented at another ophthalmology clinic, seeking refractive surgery, and was denied corneal refractive surgery with LVC, due to an alleged KC topographic pattern in OU. He sought a second opinion and was referred to our clinic. We had no access to the patient’s previous examinations. In our first observation, UDVA was 20/400 in the right eye (OD) and 20/200 in the left eye (OS), while CDVA was 20/40 (-4.75) in OD and 20/20 (-2.50/-0.75 x 75^o^) in OS. There was no personal or family history of corneal ectatic diseases. The patient used mostly glasses and occasionally soft CL to correct his refractive error. The slit lamp examination was unremarkable. Intraocular pressure (IOP) was 17 mmHg in OU. Fundoscopy revealed a tilted optic disc in OD and grade 1 fundus tessellation in OU [17]. There were no apparent subclinical or forme fruste keratoconus patterns in the topometric, tomographic, and biomechanical exams: (1) simulated keratometry (Sim K keratometry) results were 44.3 diopters (D) x 44.6 D @ 108.4^o^ in OD and 44.0 D x 44.7 D @ 100.5^o^ in OS; (2) thinnest pachymetry measurements were 546 µm OD and 534 µm OS; (3) maximum keratometry (Kmax) results were 44.9 D in OD and 45.2 D in OS.

**Figure 1:**
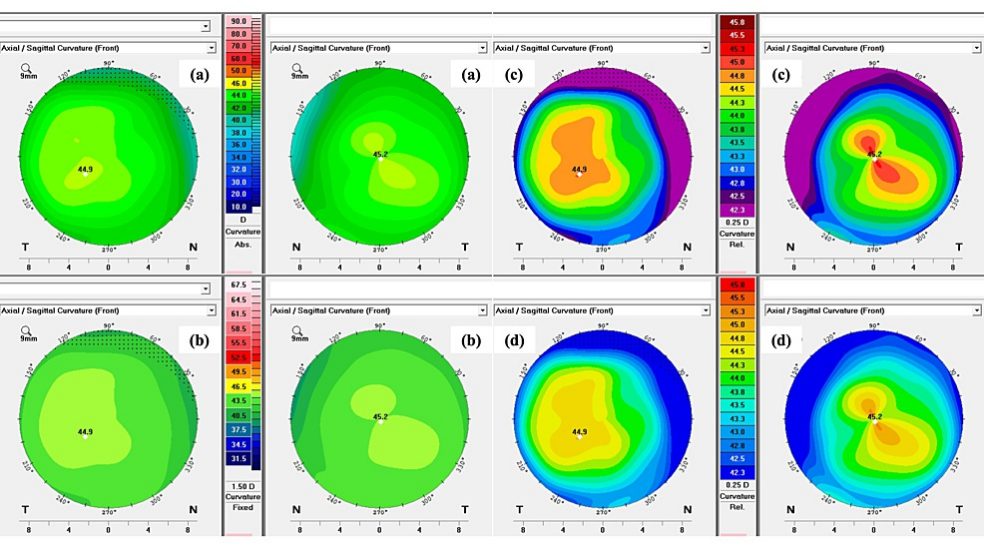
Pentacam anterior axial/sagittal curvature color maps with different scales. In (a), the Ambrósio 2 scale is displayed; in (b), the Klyce scale is displayed; in (c), the Belin scale is displayed (with a 0.25 D increment and 15-color relative scale); in (d), the Holladay scale is displayed, also with a 0.25 D increment and 15-color relative scale. There is no clear evidence of anterior axial/sagittal curvature abnormalities with the appropriate scales, though scales displayed in (c) and (d) can be misleading and result in a misdiagnosis of mild keratoconus, if not carefully interpreted.

When we altered the OCULUS Pentacam® (OCULUS Optikgeräte GmbH, Wetzlar, Germany) scale, the anterior corneal curvature presented a more suspicious pattern, though this artifact was annulled when a more appropriate scale was chosen (Figure 1).

**Figure 2:**
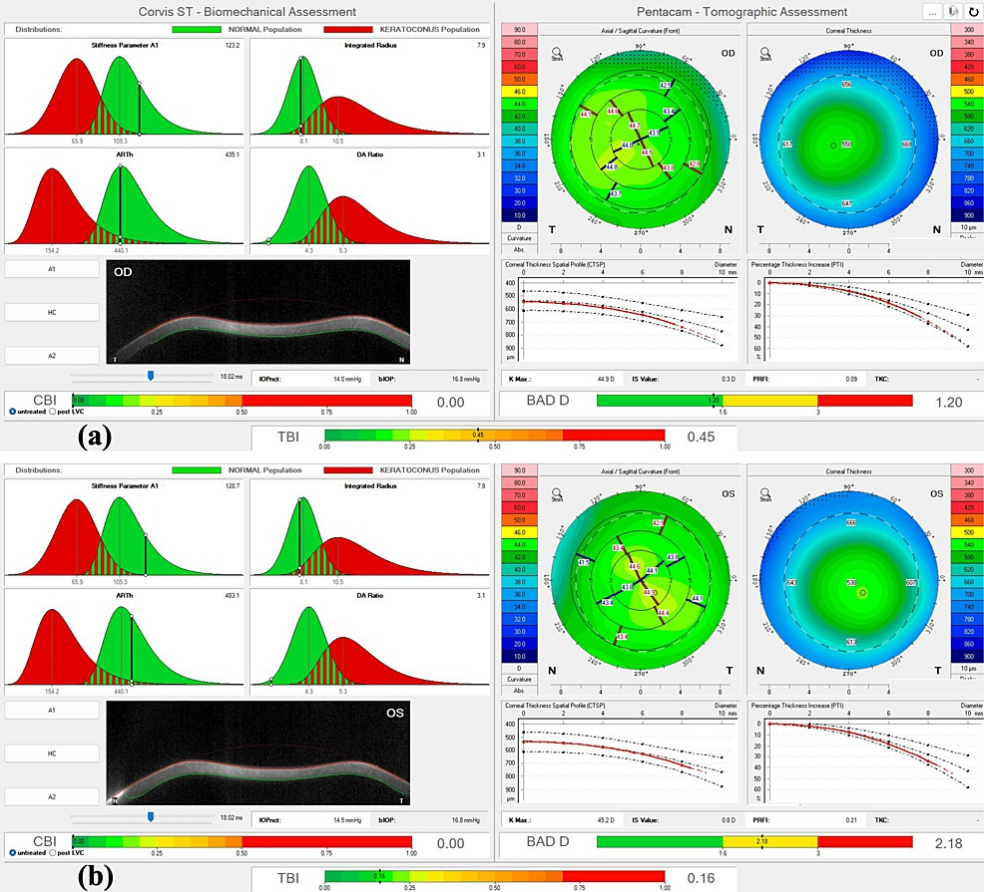
Pentacam and Corvis biomechanical/tomographic assessment (Ambrósio, Roberts & Vinciguerra (ARV)) and pre-laser vision correction assessment of the patient’s right eye (OD) and left eye (OS). The Corvis biomechanical index (CBI) and the tomographic biomechanical index (TBI) were 0.00 and 0.45 units, respectively, for the OD (a), and 0.00 and 0.6 units, respectively, for the OS (b) (indexes calculated from the data from 2013). There is no clear evidence of mild keratoconus (KC) or an ectatic disease. The Belin/Ambrósio enhanced ectasia index (BAD-D) is also depicted for both eyes, with a borderline value, but also without any clear evidence of an ectatic disease.

The Belin/Ambrósio enhanced ectasia index (BAD-D) from the OCULUS Pentacam® was 1.20 OD and 2.18 OS. The Corvis biomechanical index (CBI) and the tomographic biomechanical index (TBI) for the OCULUS Corvis® (OCULUS Optikgeräte GmbH, Wetzlar, Germany) and OCULUS Pentacam® were 0.00 and 0.45, respectively, for the OD, and 0.00 and 0.16, respectively, for the OS (Figure 2), which are values that indicate the absence of mild KC or forme fruste KC.

Furthermore, central corneal endothelial cell density was 2435 cells/mm2 in OD and 2509 cells/mm2 in OS, as analyzed with the TOMEY EM-3000® (TOMEY GmbH, Nuremberg, Germany). Axial length was 25.03 mm in OD and 24.26 mm in OS, while his anterior chamber depth was 3.65 mm in OD and 3.65 mm in OS, as measured by the IOLMaster® 500 (Carl Zeiss Meditec AG, Jena, Germany).

**Figure 3:**
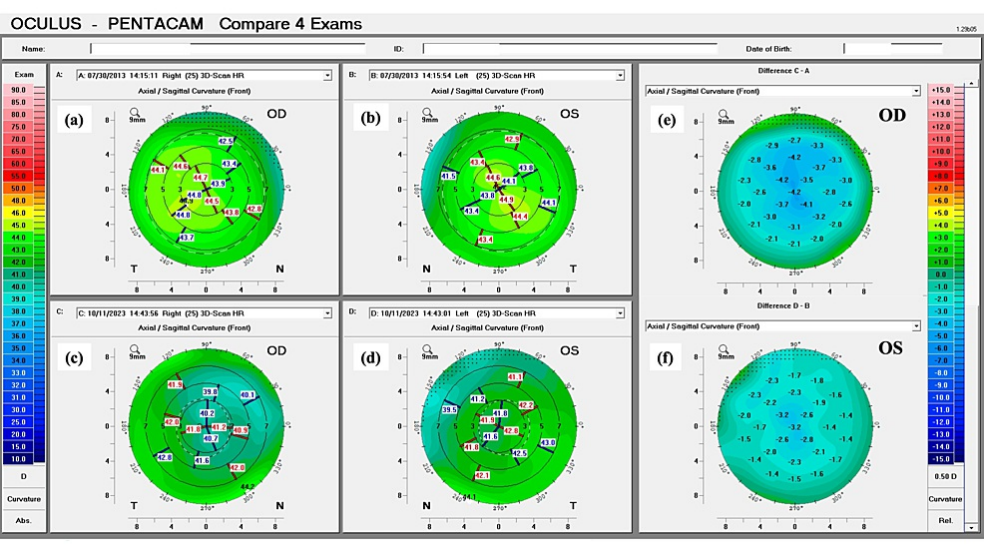
Pentacam tomographic comparison of the corneal anterior curvature pre and post-laser vision correction of the patient’s right (OD) and left (OS) eyes. (a and c): Anterior curvature maps of OD in 2013 and 2023, respectively. (b and d): Anterior curvature maps of OS in 2013 and 2023, respectively. The two panels on the right represent the difference in anterior corneal curvature between both exams, for each eye. (e and f): Note there is no evidence of the development or progression of an ectatic disease in both eyes (C–A; D–B).

**Figure 4:**
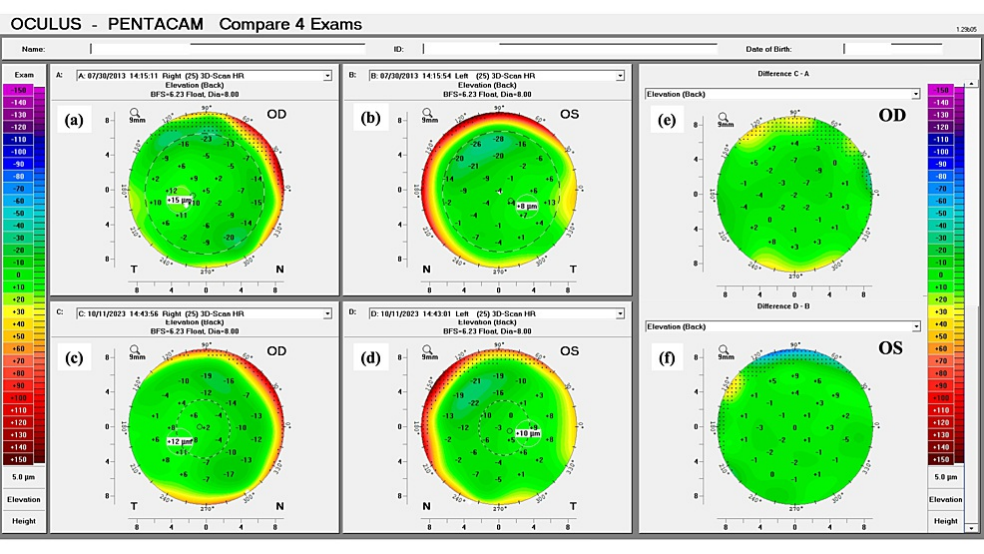
Pentacam tomographic comparison of the corneal posterior elevation pre and post-laser vision correction of the patient’s right (OD) and left (OS) eyes. (a and c): Posterior elevation maps of OD in 2013 and 2023, respectively. (b and d): Posterior elevation maps of OS in 2013 and 2023, respectively. The two panels on the right represent the difference in posterior elevation between both exams for each eye. (e and f): Note there is no evidence of the development or progression of an ectatic disease in both eyes (C–A; D–B).

**Figure 5:**
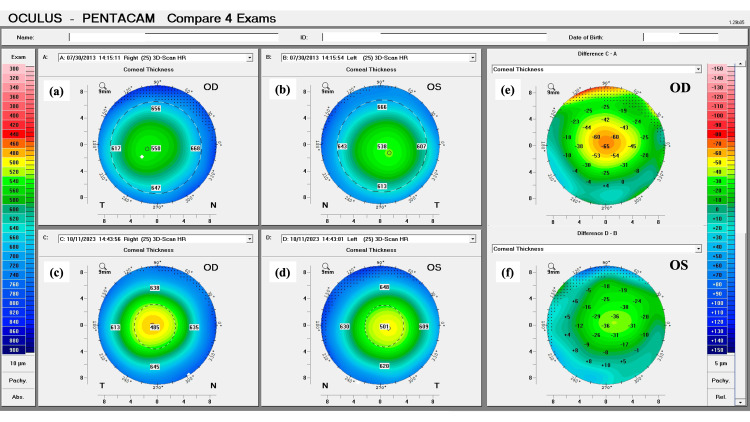
Pentacam tomographic comparison of the corneal thickness pre and post-laser vision correction of the patient’s right (OD) and left (OS) eyes. (a and c): Thickness maps of OD in 2013 and 2023, respectively. (b and d): Thickness maps of OS in 2013 and 2023, respectively. The two panels on the right represent the difference in corneal thickness between both exams, for each eye. (e and f): Note there is no evidence of the development or progression of an ectatic disease in both eyes (C–A; D–B).

The patient did not tolerate both glasses and CL, which led to a thorough discussion of the risks and benefits of refractive surgery in this particular case. The patient understood the risks and implications of being submitted to refractive surgery, in the form of PRK, without concurrent corneal cross-linking. After agreeing to the procedure, the patient underwent customized topography-guided PRK in both eyes, performed with the WaveLight® EX500 (Alcon, Fort Worth, Texas, USA). This type of ablation was used to improve the centration of the treatment near the visual axis and to obtain a more regular anterior corneal surface. Following the procedure, the patient was re-evaluated monthly in the first year after surgery, and quarterly afterward for the following year. After the first two years, the patient was re-evaluated every six months and, three years after surgery, annually. One decade after the procedure, there were no changes in the anterior corneal curvature, posterior corneal elevation, and corneal pachymetry. A stable corneal profile, with corneal flattening, was still present in both eyes (Figures 3-5).

On his last visit, the UDVA was 20/30 in the OD and 20/20 in the OS, while the CDVA was 20/25 (-0.25/-0.75 x 100°) in the OD and 20/20 (-0.25/-0.25 x 80°) in the OS. The biomechanical/tomographic assessment (Ambrósio, Roberts & Vinciguerra (ARV)) post-LVC from OD and OS demonstrated a post-LVC CBI of 0.00 in the OD and 0.00 in the OS (both normal values), as well as a BAD-D score of 2.82 in the OD and 2.35 in the OS (Table 1), which when interpreted with the rest of the data, do not indicate the development or even an increased risk of post-LVC corneal ectasia.

**Table 1:** Refractive, topographic, tomographic, and biomechanical parameters evolution from 2013 (before undergoing laser vision correction (LVC) with photorefractive keratectomy) to 2023 (10-year follow-up). There is no clear evidence of keratoconus (KC) in 2013 and there is also no evidence of development or increased risk of post-LVC corneal ectasia throughout follow-up in this patient who was initially misdiagnosed with mild KC. N/A: Not available; OD: right eye; OS: left eye. The tomographic biomechanical index cannot be calculated for eyes that have undergone LVC.

Parameter	2013		2023	
	OD	OS	OD	OS
Manifest refraction spherical equivalent	-4.75 D	-3.125 D	-0.75	-0.50
Simulated keratometry	44.3 D x 44.6 D @ 108.4^o^	44.0 D x 44.7 D @ 100.5^o^	40.4 D x 41.4 D @ 9.7^o^	41.7 D x 42.2 D @ 155.7^o^
Belin-Ambrósio Enhanced Ectasia Display (BAD-D)	1.20	2.18	2.82	2.35
Corvis Biomechanical Index (CBI)	0.00	0.00	0.00 (post-LVC)	0.00 (post-LVC)
Tomographic Biomechanical Index (TBI)	0.45	0.16	N/A	N/A

## Discussion

This case highlights the importance of performing multimodal eye imaging to increase diagnostic accuracy and choose the best individual treatment strategy for each patient. In this particular case, tomographic and biomechanical corneal evaluation was essential to our final decision to perform a customized topography-guided PRK in a patient with a suspect topography that led to the initial misdiagnosis of mild KC. This was a successful case of LVC, with no postoperative ectasia, as demonstrated by our 10-year follow-up. Interestingly, corneal tomographic and biomechanical assessments do not substitute the topographic evaluation, but instead provide additional information that is vital for the final decision. Our treatment strategy was in accordance with Tamayo et al.’s criteria for customized PRK in mild (fruste) KC [18]. These criteria include the following: (1) age over 26 years; (2) maximum keratometry < 56.00 D; (2) CCT > 430 µm; (4) manifest astigmatism < 5.0 D; (5) UDVA better than 20/400; (6) no scars or haze in the visual axis; (7) RSB > 350 µm; and (8) absence of a very inferiorly decentered cone [18]. The authors advocate that, if these criteria are followed, surface ablation, in the form of PRK, is an adequate therapeutic option for selected KC cases, particularly forms of mild KC with poor CDVA with glasses and CL intolerance, as long as there is no other contraindication for refractive surgery [18]. One should remember that a phakic intraocular lens is also a valid alternative for borderline cases with mild KC and/or moderate susceptibility for ectasia progression. Nonetheless, in this particular case, considering the patient’s refractive error and the level of required correction, advanced surface ablation was considered a valid approach.

Successful cases of PRK in milder forms of KC have been reported in multiple studies for quite some time now [12,19,20]. In 2006, Koller et al. reported the outcomes of topography-guided surface ablation in 11 eyes of eight CL-intolerant patients with forme fruste KC [19]. The authors concluded that this treatment modality is a valid option to improve vision in CL-intolerant patients, given the significant improvement of manifest refractive error, corneal irregularity, and visual aberrations, such as ghosting [19]. Posteriorly, in 2009, Chelala et al. reported the visual outcomes of PRK in 119 eyes from 72 patients who presented mild to moderate forms of KC. The authors determined that PRK was an effective and safe therapeutic option for these cases, significantly enhancing UDVA in cases of mild KC associated with low refractive errors, as was the case of our patient [12].

Performing PRK in patients with atypical topography is not devoid of risks. The surgeon must remember the possibility that these patients may already present latent corneal instability, which can aggravate following surface ablation, with consequent ectasia development or progression. Classically, in eyes with corneal instability and ectasia, LASIK is considered more perilous than PRK [9]. Furthermore, surface LVC, in the form of PRK, has successfully improved UDVA in cases of topographic irregularities, with a moderately low rate of complications. In the study by Tambe et al., 28 eyes of 23 patients with mild to moderate forms of KC underwent topography-guided PRK. The authors demonstrated the safety and efficacy of this procedure in their sample and concluded that topography-guided PRK is effective in reducing myopia and astigmatism in these patients [21]. Additionally, Khakshoor et al. reported the long-term outcomes (35 months average follow-up) of PRK in patients with mild to moderate KC (38 eyes of 21 patients; 20 eyes with grade I and 18 eyes with grade II KC), over 40 years of age, with a residual CCT of ≥400 µm. The authors reported a significant improvement in UDVA, CDVA, spherical equivalent, cylindrical power, and keratometric readings. There were no cases of KC progression or postoperative ectasia [22]. Thus, the authors suggested that a residual CCT of 400 µm might be sufficient to avoid iatrogenic KC progression, as opposed to the 450 µm cut-off value that is classically respected [12,14]. Nevertheless, there are also reports of corneal ectasia after PRK in susceptible patients. Randleman et al. reported two cases of bilateral corneal ectasia after PRK. In their series, one patient manifested early KC preoperatively, while the second patient presented a suspicious family history for KC, with a brother who required bilateral corneal transplantation at a young age [23]. Cornea ectasia after PRK can also develop multiple years after the procedure. Kim et al. reported a case of prominent corneal ectasia that developed nine years after PRK, with a residual CCT of 456 µm [24].

More importantly, one must remember to perform additional characterization of the tomographic and biomechanical properties of the patient’s eyes. The tomographic and biomechanical properties of the cornea are of paramount importance to the final therapeutic decision [25,26]. These diagnostic strategies improve the ability to detect early forms of disease (KC) and allow the identification of patients with a more significant risk for ectasia progression after LVC [26]. Therefore, current multimodal refractive imaging should involve multiple different technologies [27]. This diagnostic strategy will result in a more conscious therapeutic decision, minimizing iatrogenic ectasia, as well as decreasing the number of patients who are incorrectly denied refractive surgery, as was the patient presented in this study.

## Conclusions

In selected cases of suspect or atypical topography, surface ablation may be a reasonable therapeutic option, particularly if there is no clear evidence of mild or forme fruste KC in further preoperative multimodal imaging. Patient education is fundamental, as is complete multimodal corneal imaging. LVC, particularly in the form of PRK, is a valid therapeutic option for selected patients with low tolerance to glasses and CL, who understand and accept the risk of development or progression of corneal ectasia and accompanying further therapeutic procedures that may be required.
